# Chinese herbal medicine for recurrent aphthous stomatitis: a protocol for systematic review and meta-analysis

**DOI:** 10.1097/MD.0000000000013681

**Published:** 2018-12-14

**Authors:** Ying Zhang, Kwan-him Ng, Chih-yu Kuo, Dong-jie Wu

**Affiliations:** a Department of Geriatrics, the Third Affiliated Hospital of Zhejiang Chinese Medical University, Hangzhou, China; b School of Chinese Medicine, The University of Hong Kong, Pokfulam, Hong Kong; c LONGHUA Hospital, Shanghai University of Traditional Chinese Medicine, Shanghai; d Graduate School, Zhejiang Chinese Medical University, Hangzhou; e Clinical Research Unit, Longevity All Clinic, Quzhou, Zhejiang, China.

**Keywords:** herbal medicine, oral diseases, randomized controlled trials, recurrent aphthous stomatitis, systematic review

## Abstract

**Background:**

Recurrent aphthous stomatitis (RAS) is the most frequent form of oral ulceration, characterized by recurrent oral mucosal ulceration in an otherwise healthy individual. This study was designed to evaluate the efficacy and safety of Chinese herbal medicine for recurrent aphthous stomatitis.

**Methods:**

Five databases will be searched from inception to date, including PubMed, Cochrane Library, EMBASE,CNKI, and CBM. The researchers will comprehensively screen clinical randomized trials of Chinese herbal medicine for recurrent aphthous stomatitis. The review will be conducted by 2 independent authors without time and language limitation. The risk of bias will be assessed by the Cochrane risk of bias tool.

**Results:**

Ethical approval is not required because this study is based on published papers. After peer-review, the study will be disseminated in scientific journals and conferences.

**Conclusion:**

This systematic review will provide evidence for the efficacy and safety of Chinese medicine for recurrent aphthous stomatitis.

**PROSPERO registration:**

CRD42018111955.

## 1 Introduction

Recurrent aphthous stomatitis (RAS) is the most frequent form of oral ulceration, characterized by recurrent oral mucosal ulceration in an otherwise healthy individual.^[1–3]^ At its worst, RAS can cause significant difficulties in eating and drinking. Among the general population, the incidence of RAS is 5% to 20%.^[4–6]^ The most characteristic symptom of this disease is the single or multiple painful ulcers that appear around the oral mucosa, even on the tongue and lips.^[7,8]^ According to clinical features, RAS are mainly divided into 3 types, minor RAS, which is the most common type, major RAS whose diameter is greater than 10 cm, and herpetiform RAS.^[9–11]^ The pain causes obstacles to eating and drinking, affecting the daily life of patients.^[12,13]^

Chinese herbal medicine (CHM) is a complementary alternative medicine originating in China. It is gradually being used as a way to treat diseases in Europe and the United States.^[14–16]^ As a public disease, RAS has a high incidence in China.^[17]^ Chinese clinicians have extensive experience in treating RAS, conducting a large number of clinical trials. According to the theory of traditional Chinese medicine (TCM), the cause of this disease is heart and spleen heat, yin deficiency and fire, qi and blood deficiency, often using herbal and mineral medicine as common therapy.^[18]^ In a meta-analysis published in 2015, the effect of topical CHM on recurrent oral ulcers was evaluated.^[19]^ However, despite extensive clinical practice, clinical evidence-based literature of Chinese herbal decoction for aphthous stomatitis is not sufficient. We hope to gather recent research advances to provide evidence for clinical and public health specialist.

## 2 Methods

### 2.1 Registration

This protocol has been registered with the PROSPERO registry of the University of York. The registration number is CRD42018111955.

This systematic review protocol will follow the guidelines of Preferred Reporting Items for Systematic Reviews and Meta-Analyses (PRISMA-P).

### 2.2 Eligibility criteria

CHM treatment was defined as treatment with Chinese Herbals according to TCM pattern diagnosis. We included studies that used CHM alone or in combination with western medicine (WM).

### 2.3 Search methods for identifying the studies

#### 2.3.1 Electronic sources

We systematically searched PubMed, EMBASE, and Cochrane Library, Chinese Biomedical Database, and China Hospital Knowledge Database for randomized controlled trials of CHMs combined with compared with WM or other treatment.

The searches will be combined with the medical subject headings (Mesh) and keywords of “herbal medicine” AND “recurrent aphthous stomatitis or RAS.” References such as conference documents, research projects, doctoral, and master's thesis will be manually searched.

#### 2.3.2 Study records

##### 2.3.2.1 Data management selection process and data items

**Figure 1:**
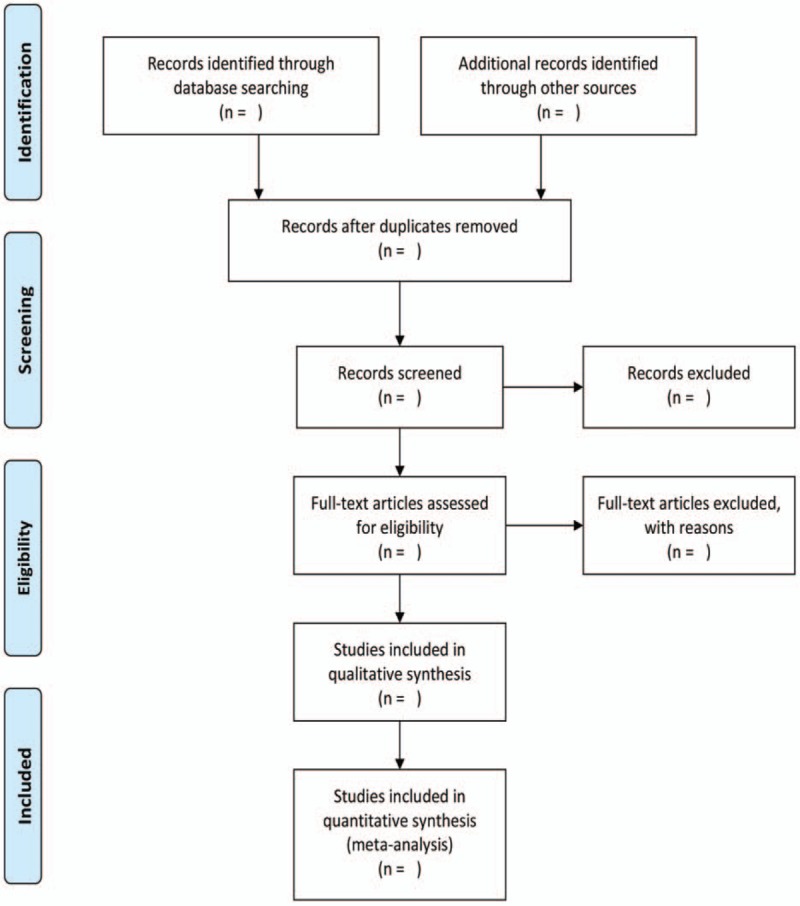
Flow diagram of study selection process.

All the articles will be confirmed by 2 independent reviewers according to the eligibility of studies, and the discrepancies will be resolved by consensus (Fig. 1). All data will be extracted and collected in a standardized template with Endnote X8 and Excel software. If the information are absent, we will contact with the corresponding authors. Standard data extraction tables include study characteristics, eligible criteria, participants’ characteristics, interventions, outcomes, follow-ups, and adverse events.

### 2.4 Data collection process

Two review authors independently extracted data in duplicate. We contacted trial authors for details of randomization, blindness, and withdrawals. We carried out risk of bias assessment on 6 domains. We followed the Cochrane Collaboration statistical guidelines and risk ratio (RR) values were to be calculated using fixed-effect models (if 2 or 3 trials in each meta-analysis) or random-effects models (if 4 or more trials in each meta-analysis).

### 2.5 Outcomes

#### 2.5.1 Primary outcomes

Primary outcome included cure rate.

#### 2.5.2 Secondary outcomes

Secondary outcomes included rate of effectiveness, rate of recurrence, healing time, adverse events.

#### 2.5.3 The risk of bias in individual studies

Two independent reviewers (NKH and KCH) will separately survey methodological quality utilizing the Cochrane risk of bias tool.^[20]^ The conflicts cannot be settled in the review will search consensus for a third author (WDJ) as required. At least 2 review authors independently assessed the risk of bias of each included study in duplicate using the Cochrane risk of bias assessment tool.^[15]^ The domains that were assessed for each included study were as follows:

(1) Sequence generation;
(2) Allocation concealment;
(3) Blinding;
(4) Completeness of outcome data;
(5) Risk of selective outcome reporting; and
(6) Risk of other potential sources of bias.

### 2.6 Data synthesis

Review Manager 5.3 (Cochrane Collaboration) is used to perform data analysis. RR is used for binary variables, and mean difference (MD) is used for continuous variables. All data are expressed with 95% confidence interval (95% CI). *I-*square (*I*^2^) and we evaluate the heterogeneity through *P* value. We use fixed effect model and random effects model based on the heterogeneity of inclusion studies.

### 2.7 Assessment of heterogeneity

The χ^2^ test was used to analyze heterogeneity. If *I*^2^ < 50%, we consider that there is no heterogeneity, and a fixed effect model is performed. If *I*^2^ > 50%, we consider that there is heterogeneity in study, using a random effects model in study. If heterogeneity exists, we conduct sensitivity analysis and subgroup analysis to detect the source of heterogeneity.

### 2.8 Assessment of reporting biases

We will use the funnel plot and the Begg test to detect publication bias of inclusion studies by Stata 14.2 software (StataCorp, College Station,TX). If *P* > .05, then there no publication bias exists in the study.

### 2.9 Analysis of subgroups or subsets

If the heterogeneity of the included studies is large, subgroup analyses will be performed to reduce heterogeneity.

### 2.10 Confidence in cumulative evidence

The quality of the research is evaluated by utilizing the Grading of Recommendations Assessment, Development and Evaluation (GRADE) approach.^[20,21]^ Utilizing the approach, the evidence is classified as high, moderate, low, very low quality based on the risk of bias, inconsistency, indirectness, imprecision, and other domains. We assume that the quality of the evidence is the highest at first and gradually decreases according to the deficiencies of the study.

## 3 Discussion

RAS is mild and self-healing diseases. However, the condition has a big impact on ordinary life of individuals because of high prevalence. The objective of this systematic review was to evaluate the efficacy and safety of CHM for the treatment of RAS.

However, this systematic review has several limitations. The quality of the study included is low and its methodology is not strict enough. The interventions of CHM also vary from study to study. High heterogeneity may also exist due to inconsistency of the included study. This is the first meta-analysis to assess the efficacy and safety of CHM for recurrent oral ulcers. We hope that our research will contribute to clinicians and public decision making.

## Author contributions

WDJ and ZY contributed to the conception of the study. The manuscript protocol was drafted by WDJ and revised by NKH. The search strategy was developed by all the authors and will be performed by WDJ and KCH, who will also independently screen the potential studies, extract data from the included studies, assess the risk of bias, and complete the data synthesis. WDJ will arbitrate in cases of disagreement and ensure the absence of errors. All authors approved the publication of the protocol.

**Conceptualization:** Dongjie Wu.

**Formal analysis:** Ying Zhang, Chih-yu Kuo.

**Software:** Ying Zhang, Kwan-Him Ng, Chih-yu Kuo.

**Supervision:** Dongjie Wu.

**Writing – original draft:** Dongjie Wu.

**Writing – review & editing:** Kwan-Him Ng.

Dongjie Wu orcid: 0000-0001-5760-5615.
